# Tinea incognito due to *microsporum gypseum*

**DOI:** 10.1016/S1674-8301(10)60014-0

**Published:** 2010-01

**Authors:** Chunshui Yu, Jingguo Zhou, Jianping Liu

**Affiliations:** Department of Dermatology, the Affiliated Hospital of North Sichuan Medical College, Nanchong 637000, China

**Keywords:** tinea incognito, *Microsporum gypseum*

## Abstract

A 41-year-old woman presented with a pruritic rash on the face that was of 3 months duration. During that time, it had been successively misdiagnosed as psoriasis vulgaris, systemic lupus erythematosus, facial dermatitis at other hospitals, and had been treated with agents that included acitretin and prednisone. Finally, fungi were found in the lesions by optical microscopy, and the fungal culture was positive for *Microsporum gypseum*, and was diagnosed as a *Microsporum gypseum* infection. The lesions eventually cleared completely after 8 weeks of antifungal treatment.

A 41-year-old woman presented with a pruritic rash on the face that had been present for 3 months. During that time, she had been treated successively with 0.05% triamcinolone acetonide and econazole nitrate cream, augmented beta-methasone dipropionate cream (0.05%), and betamethasone/clotrimazole cream. The rash transiently improved with the use of these medications but flared with any attempt to discontinue treatment. Physical examination revealed a 25-cm diameter circle of erythema with central clearing, atrophic patch with telangiectasis, pustular and slightly scaly plaques with central clearing at the lateral margin of the face was visible, excoriations and mild inflammation were noted around all affected areas ***(Fig. 1)***. The rash had been successively misdiagnosed as psoriasis vulgaris, systemic lupus erythematosus, and facial dermatitis at other hospitals, and had been treated with agents that included acitretin and prednisone.

**Fig. 1:**
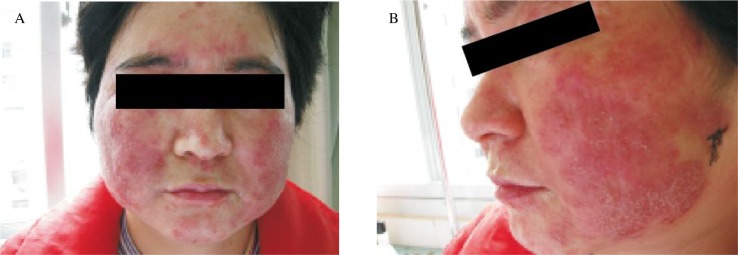
Rash in a 41-year-old women

A 10 percent potassium hydroxide examination of the lesions revealed numerous branching, septate hyphae, and the fungal culture was positive for *Microsporum gypseum*. Histopathological examination of a punch biopsy specimen from the patient's left face showed suppurative folliculitis with extension of the inflammatory process in the surrounding reticular dermis. Diagnosis was confirmed by histopathologic and mycological examination, which led to the identification of *Microsporum gypseum* (***Fig. 2***)

**Fig. 2:**
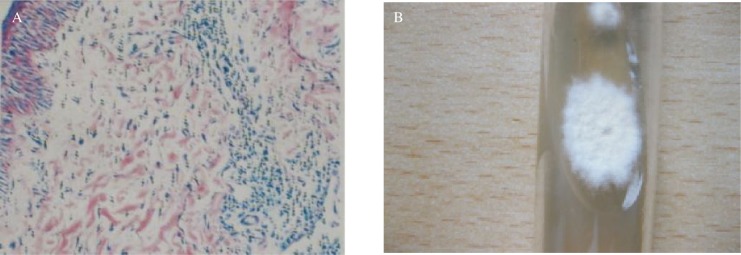
Histopathology and fungal culture of lesion. A: Histopathology; B: Fungal culture.

Topical butenafine hydrochloride cream applied twice a day to the affected area and oral itraconazole was prescribed two 100 mg capsules twice a day, Topical steroids were discontinued. After 1 week of treatment, the itching and erythema completely resolved; however, a rough and scaly plaque persisted. The eruption improved dramatically after 3 weeks ***(Fig. 3)***. and eventually cleared completely after 8 weeks of treatment.

**Fig. 3:**
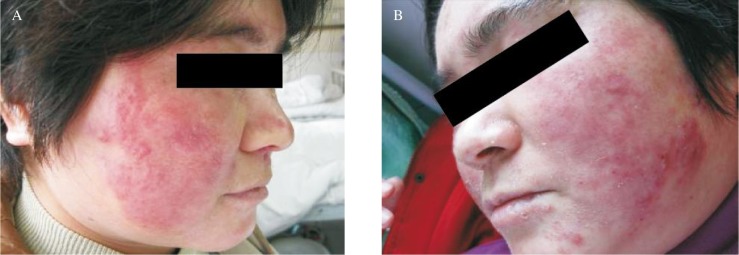
Rash after treatment. A: Three weeks after treatment; B: Four weeks after treatment.

## Discussion

“Tinea incognito” is a term used to describe a tinea infection modified by topical steroids. It is caused by prolonged use of topical steroids, sometimes prescribed as a result of incorrect diagnosis. Topical steroids suppress the local immune response and allow the fungus to grow easily. As a result, the fungal infection may take on the bizarre appearance seen in this patient[1].

Topical corticosteroids have provided a significant advance in the treatment of many dermatologic diseases since their development in the 1950s. These benefits are largely a result of the drugs' anti-inflammatory and antimitotic actions. However, the advent of higher-potency formulations has also increased the likelihood of iatrogenic side effects[2].

Local side effects include atrophy, striae, folliculitis, perioral dermatitis, and telangiectasis. The face and intertriginous areas (e.g. axilla, groin, perineum, inframammary area) are particularly susceptible to these side effects because of increased absorption through a thin stratum corneum epidermidis in these areas.

Tinea corporis classically presents as an oval or circular plaque with sharply defined, scaly, raised borders and a center that is clear or partially clear. Alternatively, the lesion may be psoriasiform, bullous, pustular, or eczematous in appearance[3]. The term tinea incognito refers to a dermatophyte infection that has been altered by use of oral or topical corticosteroids so that features typical of ringworm are masked. Very little scaling may be present. Diffuse blanching erythema with telangiectasis may be associated with scattered papules, pustules, and hyperpigmentation. In such cases, the patient's history often includes a half-moon plaque in the groin area before initiation of treatment with topical steroids.

The diagnosis of tinea incognito is simple to confirm by microscopic visualization of branching hyphae and spores typical of dermatophytes in a potassium hydroxide preparation[4]. An adequate sample of scale can be obtained from any portion of the involved patch. A particularly useful test is the fungal culture.

Treatment of tinea incognito requires cessation of all topical steroid use and implementation of specific antifungal treatment. A low-potency corticosteroid may be used briefly to avoid the flare often associated with abrupt cessation of a potent steroid. Patients should be warned of this possibility so they do not reinstitute use of topical steroids on their own.
